# Mucin 5AC expression is common but unrelated to tumor progression in pancreatic adenocarcinoma

**DOI:** 10.1177/03946320221106504

**Published:** 2022-06-28

**Authors:** Sebastian Dwertmann Rico, Franziska Büscheck, David Dum, Andreas M Luebke, Martina Kluth, Claudia Hube-Magg, Andrea Hinsch, Doris Höflmayer, Daniel Perez, Jakob R Izbicki, Michael Neipp, Hamid Mofid, Thies Daniels, Christoph Isbert, Christoph Fraune, Katharina Möller, Anne Menz, Christian Bernreuther, Patrick Lebok, Till Clauditz, Guido Sauter, Ria Uhlig, Waldemar Wilczak, Ronald Simon, Stefan Steurer, Eike Burandt, Andreas Marx, Till Krech

**Affiliations:** 1 Institute of Pathology, 37734 University Medical Center Hamburg-Eppendorf, Hamburg, Germany; 2 General, Visceral and Thoracic Surgery Department and Clinic, 37734 University Medical Center Hamburg-Eppendorf, Hamburg, Germany; 3 General, Vascular and Visceral Surgery Clinic, 40652 Itzehoe Medical Center, Itzehoe, Germany; 4 General, Visceral Thoracic and Vascular Surgery Clinic, Regio Clinic Pinneberg, Pinneberg, Germany; 5 General, Visceral and Tumor Surgery Clinic, 39611 Albertinen Hospital, Hamburg, Germany; 6 Department of General, Gastrointestinal and Colorectal Surgery, Amalie Sieveking Hospital, Hamburg, Germany; 7 Department of Pathology, Academic Hospital Fuerth, Fuerth, Germany; 8 Institute of Pathology, Clinical Center Osnabrueck, Osnabrueck, Germany

**Keywords:** mucin 5AC, pancreatic cancer, immunohistochemistry, tissue microarray

## Abstract

**Introduction:** Mucin 5AC (MUC5AC) belongs to the family of secreted gel-forming mucins. It is physiologically expressed in some normal mucin producing epithelial cells but also in pancreatic, ovarian, and colon cancer cells. The role of MUC5AC expression in cancer is not fully understood. This study was designed to explore the role of MUC5AC for pancreatic cancer progression, its association to microsatellite instability, and its diagnostic utility. **Methods:** Mucin 5AC expression was studied immunohistochemically in a tissue microarray (TMA) from 532 pancreatic cancers, 61 cancers of the ampulla Vateri, six acinar cell carcinomas and 12 large sections of pancreatitis. **Results:** Mucin 5AC staining was interpretable in 476 of 599 (79%) arrayed cancers. Staining was completely absent in normal pancreas and pancreatitis, but frequent in pancreatic cancer. Membranous and cytoplasmic MUC5AC expression was most common in pancreatic adenocarcinomas (71% of 423), followed by carcinomas of the ampulla Vateri (43% of 47), and absent in six acinar cell carcinomas. Mucin 5AC expression was unrelated to tumor phenotype (tumor stage, tumor grade, lymph node, and distant metastasis), and microsatellite instability in ductal adenocarcinomas and carcinomas of the ampulla Vateri. **Conclusion:** Our study indicates that MUC5AC is an excellent biomarker for pancreatic cancer diagnosis, especially to support the sometimes-difficult diagnosis on small biopsies. Mucin 5AC expression is unrelated to pancreatic cancer aggressiveness.

## Introduction

With almost as many deaths (432,000) as reported cases (459,000), pancreatic cancer was the seventh leading cause of cancer-related death worldwide in the year 2018, although it does not belong to the 10 most frequent cancer types.^1^ The poor prognosis of pancreatic cancer results from the paucity of early symptoms and consequently a late diagnosis of locally advanced or metastatic cancers for most patients. Radical surgical removal of the tumor followed by adjuvant chemotherapy represents the only potentially curative treatment. In recurrent or metastatic disease, chemotherapeutic options include gemcitabine, nab-paclitaxel, and a combination of fluorouracil-leucovorin-irinotecan-oxaliplatin (FOLFIRINOX).^2^ Targeted therapies such as immune checkpoint inhibitors or cancer-related proteins are rarely used in these patients. Only in the rare microsatellite instable (MSI) pancreatic carcinomas, the PD-1 inhibitor pembrolizumab can be applied based on a study showing positive response in MSI cancers irrespective of tumor origin.^3^

Mucin 5AC (MUC5AC) is of particular interest in pancreatic cancer as it is aberrantly expressed in a large fraction of these cancers. A recent study has shown that serum measurement of MUC5AC may be useful for early detection of pancreatic cancer.^4^ Mucin 5AC is one of several related secreted gel-forming glycoprotein called mucins,^5,6^ which is normally expressed in mucus producing cells of stomach, lung, and uterine cervix.^7–9^ Pathological neo-expression of MUC5AC was reported from pancreatic carcinoma and other cancers, including ovarian, appendiceal, and colorectal carcinomas.^10^ Mucin 5AC plays a role for protection and lubrication of the epithelial surface and may also contribute to cell growth, carcinogenesis, and metastasis.^11^ Moreover, MUC5AC neo-expression has been linked to MSI in colorectal and ovarian cancers.^12^ In pancreatic cancer, associations of MUC5AC expression with cancer phenotype and prognosis has earlier been studied in cohorts of 40–134 cancers and yielded controversial results.^13–15^ Yamazoe et al. reported a relationship between MUC5AC and unfavorable tumor parameters,^14^ while the other two studies did not find associations of MUC5AC and cancer phenotype.^13,15^ It is likely that small samples numbers contributed to the discrepant findings.

It was, thus, the aim of this study to analyze a large sample set to better understand the relationship of MUC5AC expression and parameters of cancer aggressiveness, and to determine whether MUC5AC expression might be linked to MSI in pancreatic cancer. For this purpose, a cohort of 599 pancreatic and ampullary cancers was analyzed for MUC5AC expression by immunohistochemistry (IHC) in a tissue microarray (TMA) format.

## Material and methods

### Tissue microarray

In this retrospective study, the TMA was constructed as previously described by Kononen et al.^16^ The TMA included 532 primary pancreatic cancers, 61 primary adenocarcinomas of the ampulla Vateri, and 6 primary pancreatic acinar cell carcinomas from the Institute of Pathology of the University Medical Center Hamburg-Eppendorf (Table 1).^17^ The tumor samples were consecutively collected from patients who underwent different types of pancreatectomy at the Department of General- Visceral- and Thoracic-Surgery, University Medical Center Hamburg-Eppendorf between 1993 and 2005, and were selected for sufficient amounts of cancer cells in the paraffin block. A single 0.6 mm core per tumor was sampled for TMA construction. The database attached to the TMA contained results on MSI measured by MLH1, MSH2, PMS2, and MSH6 IHC in 519 cases from a previous study.^18^ Large sections from 12 pancreatectomy specimens from patients with pancreatitis not suffering from carcinoma were also analyzed. Local laws (HmbKHG, §12) and the local ethics committee (Ethics Commission Hamburg, WF-049/09) provided approval for TMA manufacturing and analysis of archived remnants of diagnostic tissues for research purposes (HmbKHG, §12 and Ethics Commission Hamburg, WF-049/09). All work has been carried out in compliance with the Helsinki Declaration.

**Table 1.** Characteristics of the tissue microarray cohort.

Tumor	*N* = 599
Type	
Ductal adenocarcinoma	532 (89%)
Acinar cell carcinoma	6 (1%)
Adenocarcinoma of the ampulla Vateri	61 (10%)
Stage^a^	
pT1	20 (3%)
pT2	93 (16%)
pT3	435 (73%)
pT4	49 (8%)
Grade^a^	
1	19 (3%)
2	420 (74%)
3	130 (23%)
Lymph node status^a^	
pN0	135 (23%)
pN+	461 (77%)
Distant metastasis status^a^	
pM0	474 (79%)
pM1	123 (21%)
Surgical margin status^a^	
R0	324 (58%)
R1	231 (42%)

^a^*N* varies in subcategories due to missing values.

### Immunohistochemistry

Tissue microarray sections were stained and analyzed for MUC5AC as described previously by Rico et al.^19^ In brief, primary antibody specific against MUC5AC protein (mouse monoclonal, MSVA-109, MS Validated Antibodies, Hamburg, Germany) was applied at 1:200 dilution after antigen retrieval of the tissue sections at 121°C for 5 min in pH 7.8 TRIS-EDTA buffer. Mucin 5AC staining was seen in the membrane and cytoplasm of the cancer cells and immunostaining was interpreted as follows: Negative: no staining; weak: staining intensity of 1  +  in ≤ 70% of the tumor cells or staining intensity of 2  +  in ≤ 30% of the tumor cells; moderate: 1  + in > 70%, or 2  +  in > 30% but in ≤ 70%, or 3  +  in ≤ 30% of the tumor cells; strong: 2  +  in > 70% or 3  +  in > 30% of the tumor cells. Weak, moderate, and strong staining was considered “positive.”

### Statistical analysis

Calculations were performed with JMP^®^ (SAS Institute Inc., NC, USA). Contingency tables and chi^2^-tests were performed to find associations between MUC5AC expression and MSI, histological subtypes, or clinico-pathological parameters. A *p*-value ≤ .05 was considered significant.

## Results

### Technical issues

On our TMA, 476 of 599 (79.5%) arrayed cancers were analyzable for MUC5AC IHC. Reasons for non-informative cases (*n* = 123, 20.5%) included lack of tissue samples or absence of unequivocal cancer tissue in the TMA spot.

### Mucin 5AC expression in pancreatic cancers

**Figure 1.**
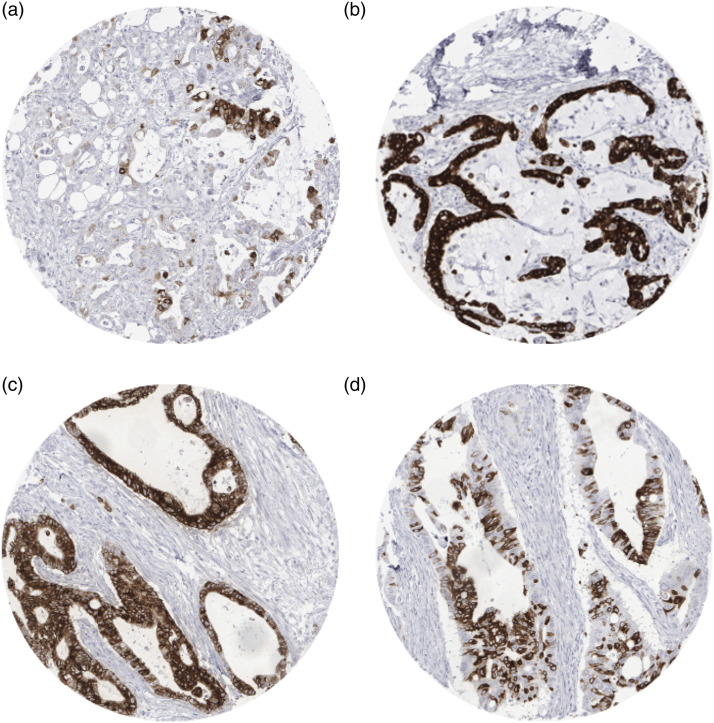
Patchy moderate to strong (A), diffuse strong (B, C), and mosaic staining pattern (D) for MUC5AC in pancreatic carcinoma.

**Figure 2.**
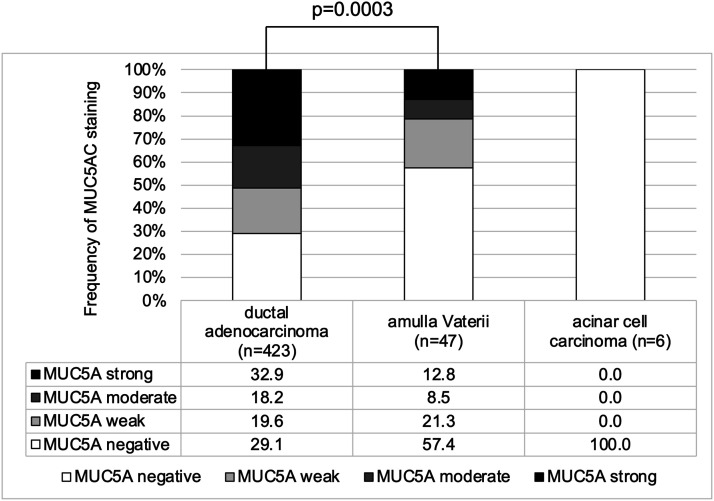
MUC5AC expression varies with histological subtype in pancreatic cancer.

Mucin 5AC staining was completely absent in normal pancreatic cells and in 12 large sections of pancreatitis. In cancers, 320 (67.2%) of the 476 interpretable samples show weak to strong membranous and cytoplasmic MUC5AC staining. The staining showed variable patterns including patchy (Figure 1(a)) and diffuse staining (Figure 1(b) and (c)). In other cancers, a variable number of positive cells were regularly distributed among negative cells (mosaic pattern; Figure 1(d)). The frequency of MUC5AC positive staining was highest in ductal adenocarcinomas of the pancreas (70.8%; *n* = 423), followed by adenocarcinomas of the ampulla Vateri (42.6%; *n* = 47; *p* = .0003 Figure 2). Mucin 5AC immunostaining was not seen in acinar cell carcinoma of the pancreas (*n* = 6).

### Mucin 5AC expression and cancer phenotype

Statistical associations were not seen between MUC5AC staining and clinico-pathological parameters, neither in the analysis of ductal adenocarcinomas of the pancreas (*p* > .07; Table 2) nor of cancers of the ampulla Vateri (*p* > .1; Table 3). Mucin 5AC staining was also unrelated to MSI in ductal adenocarcinomas (*p* = .4717; Table 2), but the relevance of this finding was limited by the small number of MSI cancers (*n* = 3).

**Table 2.** MUC5AC expression and phenotype of pancreatic ductal adenocarcinoma.

Ductal adenocarcinomas			MUC5AC (%)				
		*N*	Negative	Weak	Moderate	Strong	*p*
Total		423	29.1	19.7	18.2	32.9	
Tumor stage	pT1	12	8.3	25.0	16.7	50.0	.8119
	pT2	61	32.8	18.0	14.8	34.4	
	pT3	319	29.2	20.4	18.5	32.0	
	pT4	28	28.6	14.3	21.4	35.7	
Tumor grade	1	12	58.3	0	16.7	25.0	.0758
	2	297	26.3	21.9	18.5	33.3	
	3	91	35.2	15.4	16.5	33.0	
Lymph node status	pN0	88	25.0	25.0	14.8	35.2	.3958
	pN+	332	30.1	18.4	19.0	32.5	
Distant metastasis	pM0	334	28.7	21.0	17.1	33.2	.5067
	pM1	87	31.0	14.9	21.8	32.2	
Surgical margin status	R0	212	26.9	19.8	21.7	31.6	.3424
	R1	174	32.2	20.7	14.9	32.2	
Microsatellite status	Stable	384	28.9	19.5	18.5	33.1	.4717
	Unstable	3	33.3	33.3	33.3	0

**Table 3.** MUC5AC expression and phenotype of adenocarcinoma of the ampulla of Vateri.

Ampulla Vateri			MUC5AC (%)				
		*N*	Negative	Weak	Moderate	Strong	*p*
Total		47	57.4	21.3	8.5	12.8	
Tumor stage	pT1	2	50.0	50.0	0	0	.5421
	pT2	13	69.2	7.7	15.4	7.7	
	pT3	20	45.0	30.0	10.0	15.0	
	pT4	12	66.7	16.7	0	16.7	
Tumor grade	1	2	0	0	50.0	50.0	.1655
	2	32	56.3	21.9	9.4	12.5	
	3	13	69.2	23.1	0	7.7	
Lymph node status	pN0	9	66.7	11.1	0	22.2	.3671
	pN+	38	55.3	23.7	10.5	10.5	
Distant metastasis	pM0	38	52.6	23.7	7.9	15.8	.2611
	pM1	9	77.8	11.1	11.1	0	
Surgical margin status	R0	43	58.1	18.6	9.3	14.0	.6498
	R1	3	66.7	33.3	0	0	
Microsatellite status	Stable	44	56.8	20.5	9.1	13.6	—
	Unstable	0	—	—	—	—	

## Discussion

The results of our study demonstrate that MUC5AC expression is more frequent in ductal pancreatic adenocarcinoma (71% of 423 cancers) than in carcinomas of the ampulla Vateri (43% of 47 cancers). The frequency for pancreatic adenocarcinoma in our study is somewhat lower than in previous studies showing MUC5AC expression in 85% of 134 14 and 90% of 20 ductal adenocarcinomas.^20^ The 43% MUC5AC positivity seen for carcinomas of the ampulla Vateri is within the range of the results from earlier studies describing MUC5AC expression in 5%–62% in 6–90 evaluated cases.^21–27^ Slightly discrepant results from IHC studies are to be expected as these studies used different antibodies, IHC protocols, and cut-off levels for defining MUC5AC positivity. For example, a higher antibody dilution can be expected to result in a lower sensitivity and, consequently, in a lower fraction of positive cancers. The same applies for higher thresholds, for example, if tumors are considered positive only when a certain fraction of tumor cells (e.g., ≥ 10% or ≥ 20%) shows staining. In line with data from our study, MUC5AC expression was found to be absent in non-neoplastic tissues^14,28^ and pancreatitis^28^ in all published studies. Together with reports describing high rates of MUC5AC expression in intraductal papillary mucinous neoplasm (IPMN), a common precursor lesion of pancreatic adenocarcinoma, these findings are all consistent with a role of MUC5AC neo-expression during pancreatic cancer development.^20,29–31^

Functional in vitro and in vivo studies have consistently suggested a direct impact of MUC5AC expression on cell growth, proliferation, invasion, migration, apoptosis, and development of metastasis in pancreatic,^14,32,33^ colorectal,^34,35^ and lung cancer cell lines^36^ as well as in mouse models.^34,37^ In one study, the authors did not find differences in cell survival, proliferation, and cell morphology between siRNA-mediated knockdown cells and MUC5AC expressing cells but identified decreased tumor development and progression in a MUC5AC knockdown mouse model. Based on an increased B-lymphocyte infiltration of cancers in the MUC5AC knockdown mice, these authors suggested that MUC5AC neo-expression on the surface of pancreatic cancer cells may aid cancer cells to escape from anti-tumor effects of the immune system.^37^ This concept is also supported by data published by Hoshi et al., providing functional evidence for MUC5AC suppressing antitumor effects of neutrophils.^32^

The fact that MUC5AC expression did not show any association with the phenotype in the subsets of pancreatic and ampulla Vateri cancers, including tumor stage, tumor grade as well as lymph node and distant metastasis in our study, rather argues against a clinically significant impact of MUC5AC on cancer aggressiveness. This is in line with two earlier studies also failing to find associations between MUC5AC expression and pancreatic tumor phenotype.^13,15^ One other study investigating 134 patients found a link between high MUC5AC expression and high tumor grade, presence of lymph node metastasis, and venous invasion,^14^ and one study on ampulla Vateri cancers reported that MUC5AC expression was not only strongly associated to the pancreato-biliary phenotype, but also correlated with poor clinical outcome,^38^ however. Of note, the few studies investigating the clinical relevance of MUC5AC expression in other cancer types have also led to discrepant findings. High MUC5AC expression was linked to favorable tumor parameters in gastric and ovarian cancer,^39^ unrelated to tumor phenotype in breast and colorectal cancer,^19,40–41^ and linked to an unfavorable phenotype in lung cancers.^42^ Based on these findings, it cannot be excluded, that the biological role of MUC5AC expression in cancer cells might be dependent on the tumor type.

That MUC5AC expression was detectable in more than 70% of pancreatic adenocarcinomas, but completely absent in normal and inflamed pancreatic tissue, suggests a high diagnostic utility of MUC5AC IHC. This is supported by a study in which all IPMNs analyzed were shown to express MUC5AC 20. Elevated MUC5AC levels are also detectable by enzyme-linked immunosorbent assays in the serum of pancreatic cancer patients.^43^ In one study, the combined measurement of serum levels of MUC5AC and CA19-9—the best-established diagnostic serum marker for pancreatic cancer—showed higher specificity and sensitivity than CA19-9 alone in differentiating pancreatic cancer from normal tissue, benign neoplasms and pancreatitis.^4^ Measurement of patient’s MUC5AC serum levels could not only be useful for potential early diagnosis but also serve for monitoring of recurrence and response to therapy.

Mucin 5AC is the molecular target of ensituximab (Neo-102), a chimeric monoclonal antibody that binds to an aberrantly glycosylated cancer-associated MUC5AC variant and activates the immune system to exert a cytotoxic T-lymphocyte response.^44^ In a phase I study of pancreatic cancer patients preselected for MUC5AC expression, a favorable toxicity profile was found for ensituximab.^44^ Ensituximab resulted in stable disease in 21% of 56 patients with heavily pretreated refractory colorectal cancers and was well tolerated in a Phase II clinical trial.^45^ Of note, MUC5AC positivity was defined as staining in ≥20% of tumor cells in these latter studies. If the same criteria are applied to our study, MUC5AC is positive in at least 55% of all pancreatic cancers, suggesting that this tumor type may be an ideal application for new drugs specifically targeting MUC5AC.

A TMA with 599 tumor samples was used in this study. It is the nature of TMAs that the sample size is not calculated for a specific study, but that as many samples as possible are included to generate a platform for multiple studies and a molecular database with results from these analyses. The total number of 476 interpretable tumors for MUC5AC was sufficient to find significant differences in the MUC5AC positivity between pancreatic cancers and ampulla Vateri cancers, and to exclude significant association with parameters of tumor aggressiveness or microsatellite status within these subsets. The microsatellite status was determined by IHC and MSI-PCR in an earlier study using our TMA.^18^ The rate of 0.8% MSI positive pancreatic cancers in that study fitted well to the 0.8–1.1% MSI positive pancreatic cancers reported from studies using next generation sequencing (NGS).^46,47^ Of note, in 2019, the ESMO recommended NGS for microsatellite analysis in tumor types with low frequency of MSI and little data available on the reliability of IHC and MSI-PCR, including pancreatic, cervical, extrahepatic bile duct, prostate, non-small cell lung cancer, head and neck, anal, and kidney cancers as well as melanomas and sarcomas.^48^ Our study is an example on how TMAs can contribute to establish solid data for microsatellite status IHC in such tumor types.

## Conclusions

In summary, the results of this study show that MUC5AC is an excellent biomarker for diagnosing pancreatic cancers and may facilitate this difficult diagnosis on small biopsies. However, despite functional evidence for a cancer promoting role, MUC5AC is not associated with unfavorable clinico-pathological parameters in pancreatic cancer.
